# PERK/ATF4-dependent expression of the stress response protein REDD1 promotes proinflammatory cytokine expression in the heart of obese mice

**DOI:** 10.1152/ajpendo.00238.2022

**Published:** 2022-11-16

**Authors:** Shaunaci A. Stevens, Maria K. Gonzalez Aguiar, Allyson L. Toro, Esma I. Yerlikaya, Siddharth Sunilkumar, Ashley M. VanCleave, Jessica Pfleger, Elisa A. Bradley, Scot R. Kimball, Michael D. Dennis

**Affiliations:** ^1^Department of Cellular and Molecular Physiology, Penn State College of Medicine, Hershey, Pennsylvania; ^2^Fralin Biomedical Research Institute, Virginia Tech, Roanoke, Virginia; ^3^Division of Cardiovascular Medicine, Penn State Health Heart and Vascular Institute, Hershey S. Milton Medical Center, Hershey, Pennsylvania

**Keywords:** DDIT4, diabetes, endoplasmic reticulum stress, inflammation, obesity

## Abstract

Endoplasmic reticulum (ER) stress and inflammation are hallmarks of myocardial impairment. Here, we investigated the role of the stress response protein regulated in development and DNA damage 1 (REDD1) as a molecular link between ER stress and inflammation in cardiomyocytes. In mice fed a high-fat high-sucrose (HFHS, 42% kcal fat, 34% sucrose by weight) diet for 12 wk, REDD1 expression in the heart was increased in coordination with markers of ER stress and inflammation. In human AC16 cardiomyocytes exposed to either hyperglycemic conditions or the saturated fatty acid palmitate, REDD1 expression was increased coincident with ER stress and upregulated expression of the proinflammatory cytokines IL-1β, IL-6, and TNFα. In cardiomyocytes exposed to hyperglycemic/hyperlipidemic conditions, pharmacological inhibition of the ER kinase protein kinase RNA-like endoplasmic reticulum kinase (PERK) or knockdown of the transcription factor ATF4 prevented the increase in REDD1 expression. REDD1 deletion reduced proinflammatory cytokine expression in both cardiomyocytes exposed to hyperglycemic/hyperlipidemic conditions and in the hearts of obese mice. Overall, the findings support a model wherein HFHS diet contributes to the development of inflammation in cardiomyocytes by promoting REDD1 expression via activation of a PERK/ATF4 signaling axis.

**NEW & NOTEWORTHY** Interplay between endoplasmic reticulum stress and inflammation contributes to cardiovascular disease progression. The studies here identify the stress response protein known as REDD1 as a missing molecular link that connects the development of endoplasmic reticulum stress with increased production of proinflammatory cytokines in the hearts of obese mice.

## Graphical abstract

**Figure:**
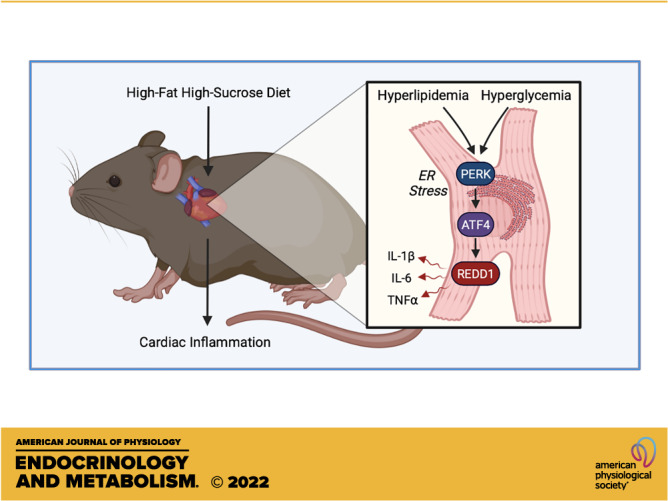

## INTRODUCTION

Type 2 diabetes and obesity are an active and rapidly expanding public health crisis, impacting nearly half of American adults. The leading cause of mortality and morbidity in patients with diabetes and obesity is heart failure (1). Diabetic cardiomyopathy (DC) is a metabolically triggered myopathic process typically characterized by left ventricular hypertrophy and fibrosis that lead to activation of the ischemic cascade resulting first in diastolic dysfunction, and later progressing to systolic dysfunction, in the absence of large vessel coronary artery disease (2). Although there have been data demonstrating that mechanical factors may contribute to the development of DC, many of the molecular mechanisms that drive cardiomyocyte dysfunction remain underexplored. At the cellular level, endoplasmic reticulum (ER) stress and inflammation are considered hallmarks of myocardial impairment, and the interplay between these stress response pathways contributes to cardiovascular disease progression (3).

The ER is an organelle that functions in protein and lipid synthesis, protein folding/maturation, and calcium homeostasis. Type 2 diabetes and obesity disrupt ER homeostasis, leading to the accumulation of misfolded and unfolded proteins (4, 5). Diets rich in fats enhance plasma levels of free fatty acids (FA) and promote FA uptake into cardiomyocytes (6). Accumulation of lipid droplets and toxic lipid intermediates such as palmitate, ceramide, and diacylglycerol triggers chronic upregulation of ER stress (7, 8), leading to cardiac dysfunction (9). In addition to hyperlipidemia, hyperglycemia also promotes ER stress in cardiomyocytes by upregulating the production of reactive oxygen species (10).

ER stress activates the unfolded protein response (UPR), which is designed to restore ER homeostasis by reducing protein synthesis and clearing unfolded/misfolded proteins. Protein kinase R (PKR)-like endoplasmic reticulum kinase (PERK), inositol-requiring enzyme-1 (IRE1), and activating transcription factor 6 (ATF6) represent three main regulatory branches of the UPR (11). In the absence of ER stress, the luminal domains of PERK, IRE1, and ATF6 are sequestered by the ER chaperone 78-kDa glucose-related protein (GRP78). Accumulation of unfolded proteins within the ER lumen causes GRP78 detachment from PERK, IRE1, and ATF6. PERK and IRE1 become activated via auto/transphosphorylation and the formation of homo-oligomers. IRE1 facilitates splicing of X-box DNA-binding protein 1 (XBP1) mRNA to generate the transcription factor XBP1s. PERK phosphorylates the α-subunit of the translation initiation factor eIF2, leading to a reduction in global rates of mRNA translation (12). However, phosphorylation of eIF2α promotes the preferential translation of the mRNA encoding activating transcription factor 4 (ATF4). ATF4 is a basic leucine zipper transcription factor that stimulates the expression of several stress response genes, including ATF3 and regulated in development and DNA damage 1 (REDD1, also known as DDIT4/RTP801) (13).

Our laboratory recently demonstrated that diabetes promotes REDD1 protein expression in both the retina (14–18) and the kidney (19), and that REDD1 deletion is sufficient to prevent diabetes-induced functional deficits in vision (20, 21), as well as renal complications (19). A similar increase in REDD1 protein expression has also been observed in the myocardial tissue of diet-induced obese rats (22); however, to our knowledge, neither the mechanism responsible for increased cardiac REDD1 expression, nor a role for REDD1 in DC pathology has been previously interrogated. A recent study using H9c2 cardiomyocytes demonstrated that REDD1 knockdown suppressed injury following oxygen/glucose deprivation followed by reperfusion (OGD/R) (23). Similarly, REDD1 knockdown reduced ischemia-reperfusion injury in hearts that were genetically manipulated to overexpress TXNIP (24). Remarkably, a protective effect of REDD1 knockdown was observed in the development of cardiac dysfunction in a murine model of doxorubicin-induced cardiomyopathy (25). Herein, we investigated the impact of a prodiabetogenic diet on REDD1 expression and inflammation in the heart. Overall, the results support a model wherein diabetes contributes to the development of inflammation in cardiomyocytes by promoting REDD1 expression via activation of a PERK/ATF4 signaling axis.

## RESEARCH DESIGN AND METHODS

### Animals

Starting at 6 wk of age, C57BL/6J mice were fed either a Teklad control diet (TD.08485) containing 13% kcal from fat, 63.3% from carbohydrates, and 19.1% from protein or a high-fat high-sucrose diet (TD.88137) containing 42% kcal from fat, 42% kcal from carbohydrates, and 15.2% from protein (Envigo, Huntington, UK) for up to 16 wk. A similar series of studies were performed on B6;129 REDD1^+/+^ and REDD1^−/−^ mice. REDD1^−/−^ mice were generated as previously described (26) and exhibit whole body REDD1 knockout. Euthanasia was carried out by isoflurane inhalation followed by dissection of the diaphragm. Cardiac tissue was collected for analysis. All procedures adhered to the National Institutes of Health Guide for the Care and Use of Laboratory Animals and were approved by the Penn State College of Medicine Institutional Animal Care and Use committee.

### Cell Culture

Human AC16 adult ventricular cardiomyocyte cells were obtained from Sigma-Aldrich (SCC109, RRID: CVCL_4U18). Cells were cultured in Dulbecco’s modified Eagle’s medium/nutrient mixture F-12 (Gibco, 11320082) supplemented with 10% fetal bovine serum (FBS, Atlas Biologicals) and 1% penicillin/streptomycin (P/S) in a humidified incubator with 5% CO_2_ at 37°C. Before use, cells were adapted to Dulbecco’s modified Eagle’s medium (DMEM, Gibco 11885092) containing 5 mM glucose for two passages. Cells were used up to passage 11. Rat H9c2(2-1) ventricular cardiomyocytes were obtained from American Type Culture Collection (ATCC) (CRL-1446) and cultured in DMEM (ATCC 30–2002) supplemented with 10% FBS and 1% P/S. ATF4^+/+^ and ATF4^−/−^ MEFs (27) were kindly provided by Drs. David Ron and Heather Harding, University of Cambridge Institute for Medical Research. As previously described (28), cells were maintained in DMEM supplemented with 10% FBS, 1% P/S, 55 µM β-mercaptoethanol (Sigma-Aldrich), and 1X nonessential amino acids (Gibco). In specific studies, cells were exposed to culture medium containing either 25 mM glucose and/or 0.5 mM palmitate or 5 mM glucose supplemented with 20 mM mannitol as an osmotic control for up to 48 h. In other studies, cells were exposed to medium containing 2 µg/mL tunicamycin (Tocris Bioscience) or 1 µM GSK2606414 (Selleck Chemicals). pLKO-shATF4 plasmids were obtained from the Penn State College of Medicine TRC1 Human Library Informatics Core (shATF4 TRC ID# TRCN0000013573). Lentivirus containing shRNA targeting ATF4 (sequence: 5′- CCGGGCCTAGGTCTCTTAGATGATTCTCGAGAATCATCTAAGAGACCTAGGCTTTTT-3′) was prepared with HEK293FT cells and used to infect AC16 cells. Puromycin (2 µg/mL) was used to select shRNA-expressing cells. Stable knockdown was verified by Western blot and qPCR. A stable REDD1 knockout AC16 cell line was generated by CRISPR/Cas9 using a previously described pLentiCRISPR v.2 construct containing a REDD1 (DDIT4) guide RNA (15). AC16 cells were incubated with viral particles, selected with puromycin, and REDD1 protein expression was evaluated.

### PCR Analysis

Total RNA was extracted with TRIzol (Invitrogen) according to the manufacture’s protocol. RNA (1 μg) was reverse transcribed using a high capacity cDNA Reverse Transcription Kit (Applied Biosystems) and subjected to quantitative real-time PCR (qRT-PCR) (QuantStudio 12 K Flex Real-Time PCR System, RRID:SCR_021098) using QuantiTect SYBR Green master mix (Qiagen). Primer sequences are listed in Supplemental Table S1. Mean cycle threshold (*C_T_*) values were determined for control and experimental samples. Changes in mRNA expression were normalized to glyceraldehyde 3-phosphate dehydrogenase (GAPDH) mRNA expression using the 2^−ΔΔCT^ calculations.

### Protein Analysis

Homogenization buffer containing 20 mM HEPES (pH 7.4), 2 mM EGTA, 50 mM NaF, 100 mM KCl, 0.2 mM EDTA-Na2, 50 mM B-glycerophosphate and supplemented with the following inhibitors, 1 mM DTT, 1 mM benzamidine, 0.5 mM sodium vanadate, and 10 µL/mL protease µinhibitor cocktail (P8340, Sigma-Aldrich) was used to homogenize cardiac tissue. Tissue homogenate was centrifuged at 10,000 *g* for 10 min at 4°C and the supernatant was collected for analysis. Tissue supernatant or lysates from cell culture were combined with sodium dodecyl sulfate (SDS) sample buffer, boiled for 5 min, and subjected to Western blot analysis. Proteins were fractionated using criterion precast 4%–20% gels (Bio-Rad Laboratories, Hercules, CA), transferred to polyvinylidene fluoride (PVDF), blocked in 5% milk in Tris-buffered saline Tween 20 (TBS-T), washed, and incubated overnight at 4°C with primary antibody (Supplemental Table S2). The next day, the PVDF membrane was washed, blocked in 5% milk TBS-T supplemented with the appropriate secondary antibody (Supplemental Table S2), and washed again. The antigen-antibody interaction was visualized with enhanced chemiluminescence clarity reagent (Bio-Rad Laboratories) using a ProteinSimple FluorChem E imaging system. Concentrations of IL-1β were determined in heart tissue homogenates using the mouse IL-1β/IL-1F2 Quantikine ELISA Kit (R&D Systems) following the manufacturer’s instructions.

### Data Analysis and Presentation

Statistical analyses were performed using GraphPad Prism software with *P* value <0.05 defined as statistically significant. Data were analyzed overall with either Student’s *t* test or one-way or two-way analysis of variance. Trend test and pairwise comparisons were conducted with the Tukey test for multiple comparisons. Figures were assembled with Adobe Illustrator.

## RESULTS

### High-Fat High-Sucrose Diet Promoted REDD1 Protein Expression, ER Stress, and Inflammation in the Heart

**Figure 1.**
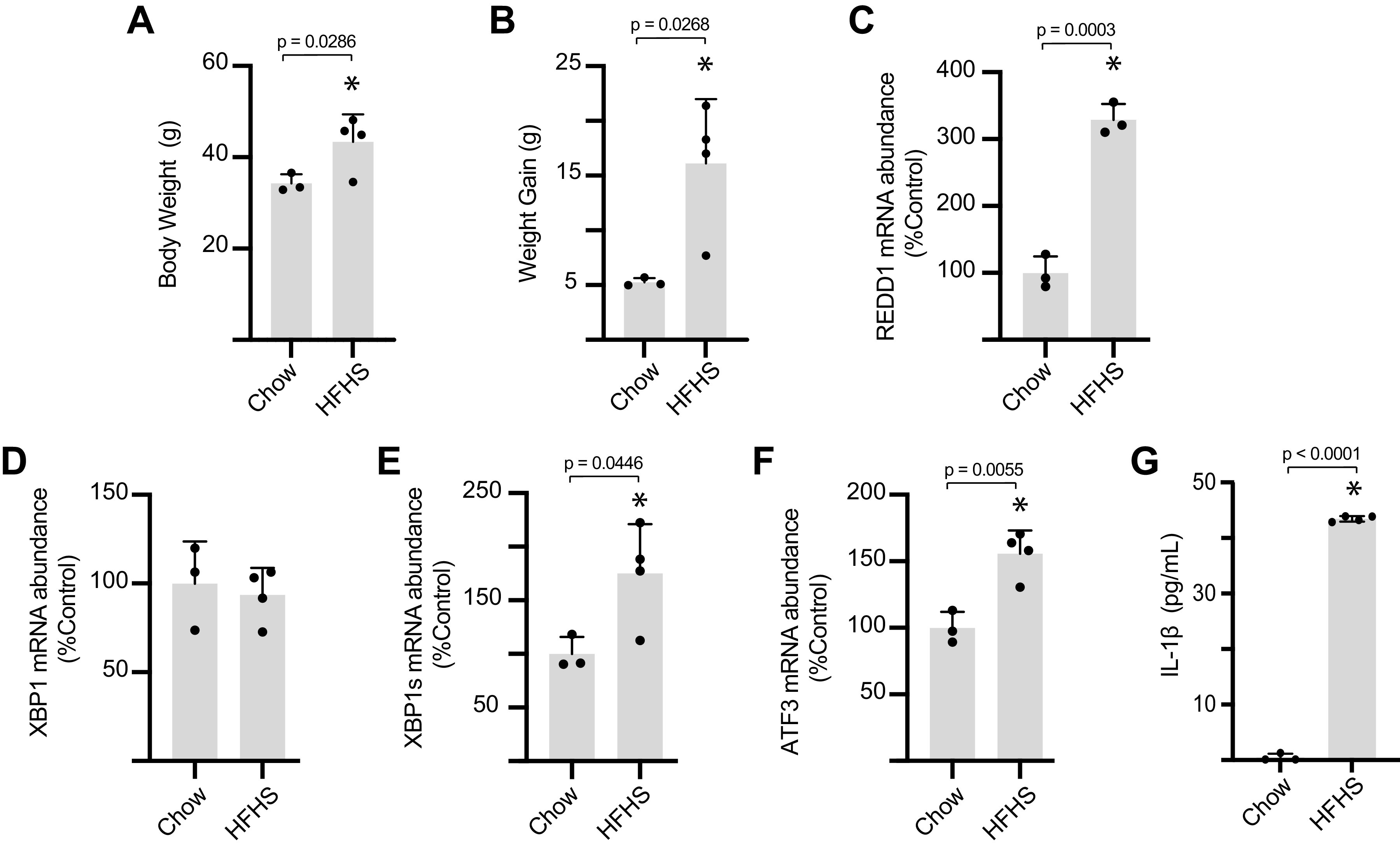
High-fat high-sucrose (HFHS) diet promotes REDD1 expression, markers of ER stress, and IL-1β expression in the heart. Mice were fed either a HFHS diet or control chow diet for 12 wk. Body weight (*A*) and weight gain (*B*) were determined. Expression of mRNAs encoding REDD1 (*C*), XBP1 (*D*), XBP1s (*E*), and ATF3 (*F*) were evaluated in cardiac tissue homogenates by qPCR. IL-1β protein expression in cardiac tissue homogenates was determined by ELISA (*G*). Results are represented as means ± SD (*n* = 3 or 4 mice). *P < 0.05 versus chow. ATF3, activating transcription factor 3; ER, endoplasmic reticulum; REDD1, regulated in development and DNA damage 1; XBP1, X-box DNA-binding protein 1.

To explore the impact of a prodiabetogenic diet on cardiac REDD1 expression, mice were fed an HFHS diet for 12 wk. Mice fed an HFHS diet exhibited increased body weight (Fig. 1*A*) and enhanced weight gain (Fig. 1*B*) as compared with littermate controls fed a chow diet. Analysis of heart tissue homogenates revealed increased expression of REDD1 in the hearts of mice fed an HFHS diet (Fig. 1*C*). To explore a potential relationship between cardiovascular expression of REDD1 and ER stress, we examined XBP1 and ATF3 mRNA abundances, as markers for IRE1 and PERK/ATF4 activation, respectively. XBP1 splicing was enhanced (Fig. 1, *D* and *E*) and ATF3 mRNA abundance was increased (Fig. 1*F*) in the hearts of mice fed an HFHS diet. Given that interleukin (IL)-1β is a central driver of the cardiovascular complications caused by diabetes and is an established target for clincal intervention (29), we also examined the relative expression of IL-1β and found it to be enhanced in cardiac tissue of mice fed an HFHS diet (Fig. 1*G*).

### ER Stress Was Sufficient to Promote REDD1 and Inflammatory Cytokine Expression in Cardiomyocyte Cultures

**Figure 2.**
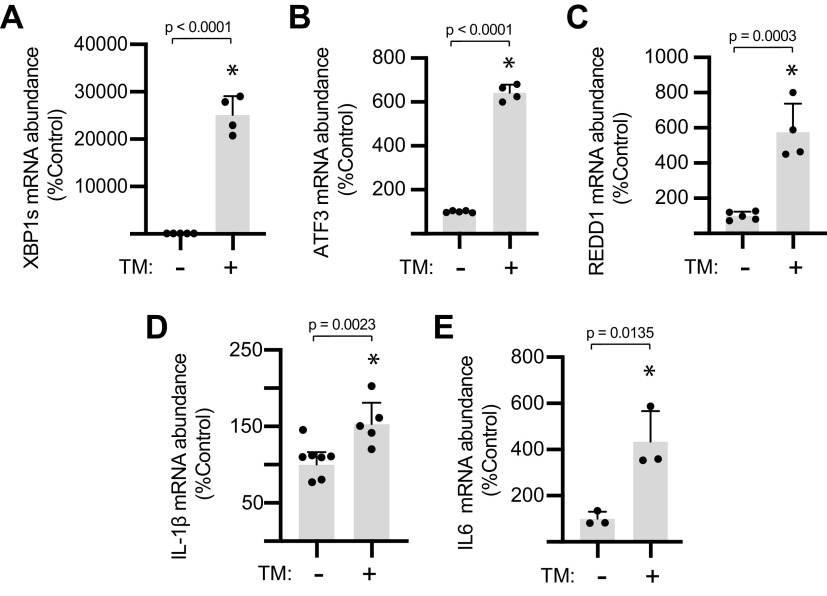
ER stress is sufficient to promote REDD1 and inflammatory cytokine expression in cardiomyocyte cultures. Human AC16 cardiomyocyte cultures were exposed to medium containing tunicamycin (TM) for 4 h, as indicated. Expression of mRNAs encoding XBP1s (*A*), ATF3 (*B*), REDD1 (*C*), IL-1β (*D*), and IL6 (*E*) was evaluated in cell lysates by qPCR. Results are represented as means ± SD (*n* = 3–5 replicates). *P < 0.05 versus without TM. ATF3, activating transcription factor 3; ER, endoplasmic reticulum; REDD1, regulated in development and DNA damage 1; XBP1, X-box DNA-binding protein 1.

To evaluate the impact of ER stress in human cardiomyocytes, we exposed human AC16 cells to tunicamycin to block N-linked glycosylation and thus activate the UPR. In support of ER stress, tunicamycin increased XBP1 splicing (Fig. 2*A*) and enhanced ATF3 mRNA abundance (Fig. 2*B*). Moreover, REDD1 mRNA abundance was enhanced by tunicamycin (Fig. 2*C*). Notably, rat H9c2 cardiomyocytes exposed to tunicamycin also exhibited increased XBP1 splicing and enhanced ATF3 and REDD1 mRNA abundance (Supplemental Fig. S1). In coordination with ER stress and upregulated REDD1 expression, AC16 cardiomyocytes exposed to tunicamycin also exhibited upregulated expression of mRNAs encoding IL-1β (Fig. 2*D*) and IL6 (Fig. 2*E*).

### ER Stress and Enhanced REDD1 Expression Manifest in Cardiomyocytes Exposed to Hyperglycemic and Hyperlipidemic Conditions

**Figure 3.**
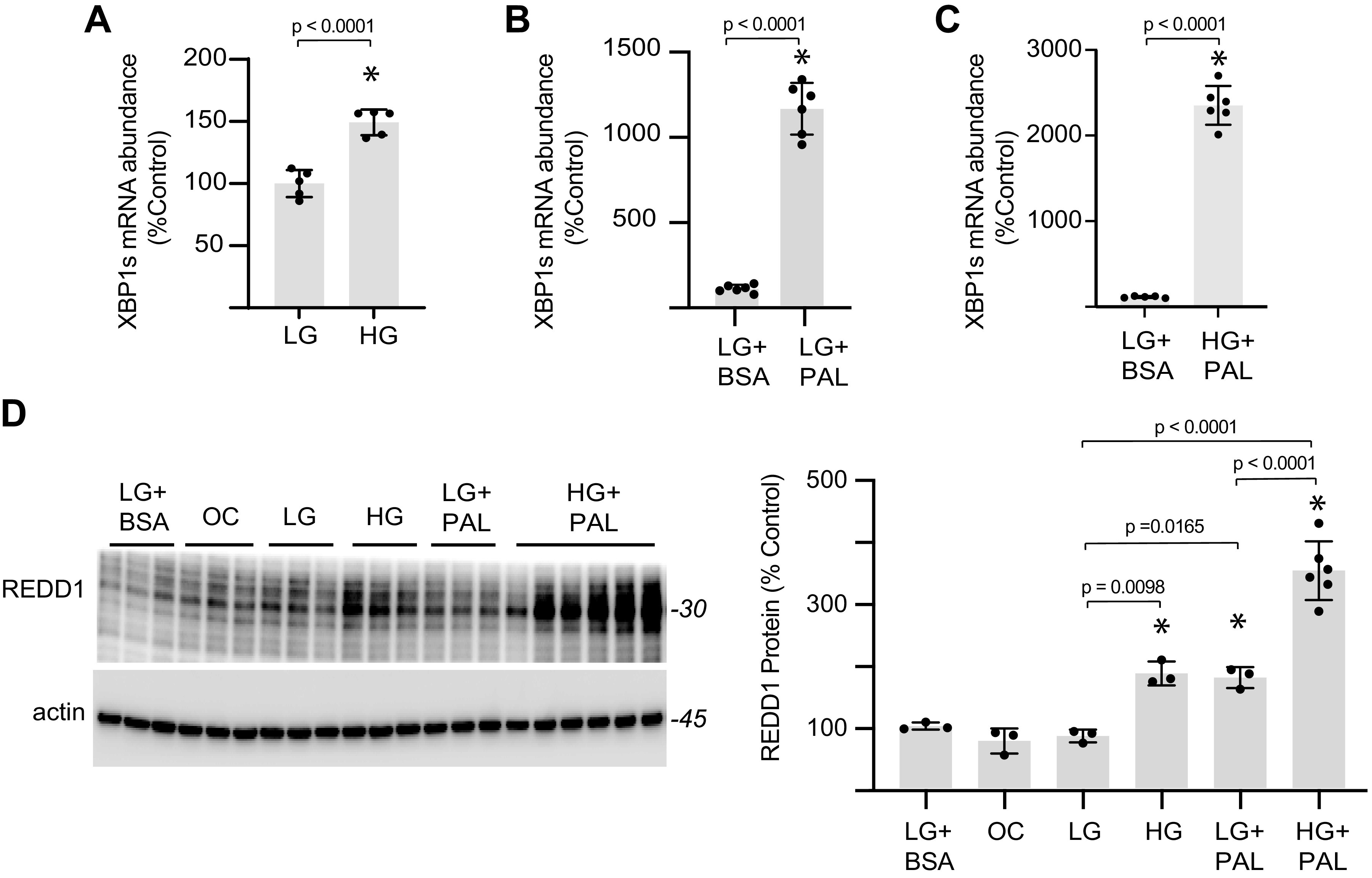
Cardiomyocytes exposed to hyperglycemic or hyperlipidemic conditions exhibit ER stress and enhanced REDD1 expression. *A*: human AC16 cardiomyocytes were exposed to culture medium containing either 5 mM (LG) or 25 mM (HG) glucose for 48 h. *B*: AC16 cells were exposed to culture medium containing either 0.5 mM BSA-conjugated palmitate (PAL) or BSA alone for 48 h. *C*: AC16 cells were exposed to culture medium containing LG and BSA or HG and PAL for 48 h. XBP1s mRNA expression was evaluated in cell lysates by qPCR. *D*; REDD1 protein expression was evaluated in AC16 cells exposed to culture medium containing either LG, HG, 5 mM glucose with 20 mM mannitol as an osmotic control (OC), BSA, or PAL. REDD1 and actin protein expression in cell lysates was assessed by Western blotting. Representative blots are shown. Protein molecular mass in kDa is shown at *right* of blots. Results are represented as means ± SD (*n* = 3–6 replicates). *P < 0.05 versus LG or LG+BSA. ER, endoplasmic reticulum; REDD1, regulated in development and DNA damage 1; XBP1, X-box DNA-binding protein 1.

The impact of hyperglycemic and hyperlipidemic conditions on AC16 cardiomyocytes was explored by exposing cells to culture medium supplemented with 25 mM glucose and/or 0.5 mM of the long-chain saturated fatty acid palmitate. Cardiomyocytes exposed to hyperglycemic conditions exhibited enhanced XBP1s mRNA abundance (Fig. 3*A*), supporting the development of ER stress. XBP1s mRNA abundance was also increased in cardiomyocytes exposed to a medium supplemented with palmitate (Fig. 3*B*), and there was an additive effect in cells exposed to both hyperglycemic conditions and palmitate (Fig. 3*C*). Similarly, REDD1 protein expression was increased in cardiomyocytes exposed to either hyperglycemic conditions and/or excess palmitate (Fig. 3*D*). Moreover, there was an additive increase in REDD1 expression when the two conditions were combined.

### PERK Was Required for Enhanced REDD1 Expression in Cardiomyocytes Exposed to Hyperglycemic and Hyperlipidemic Conditions

**Figure 4.**
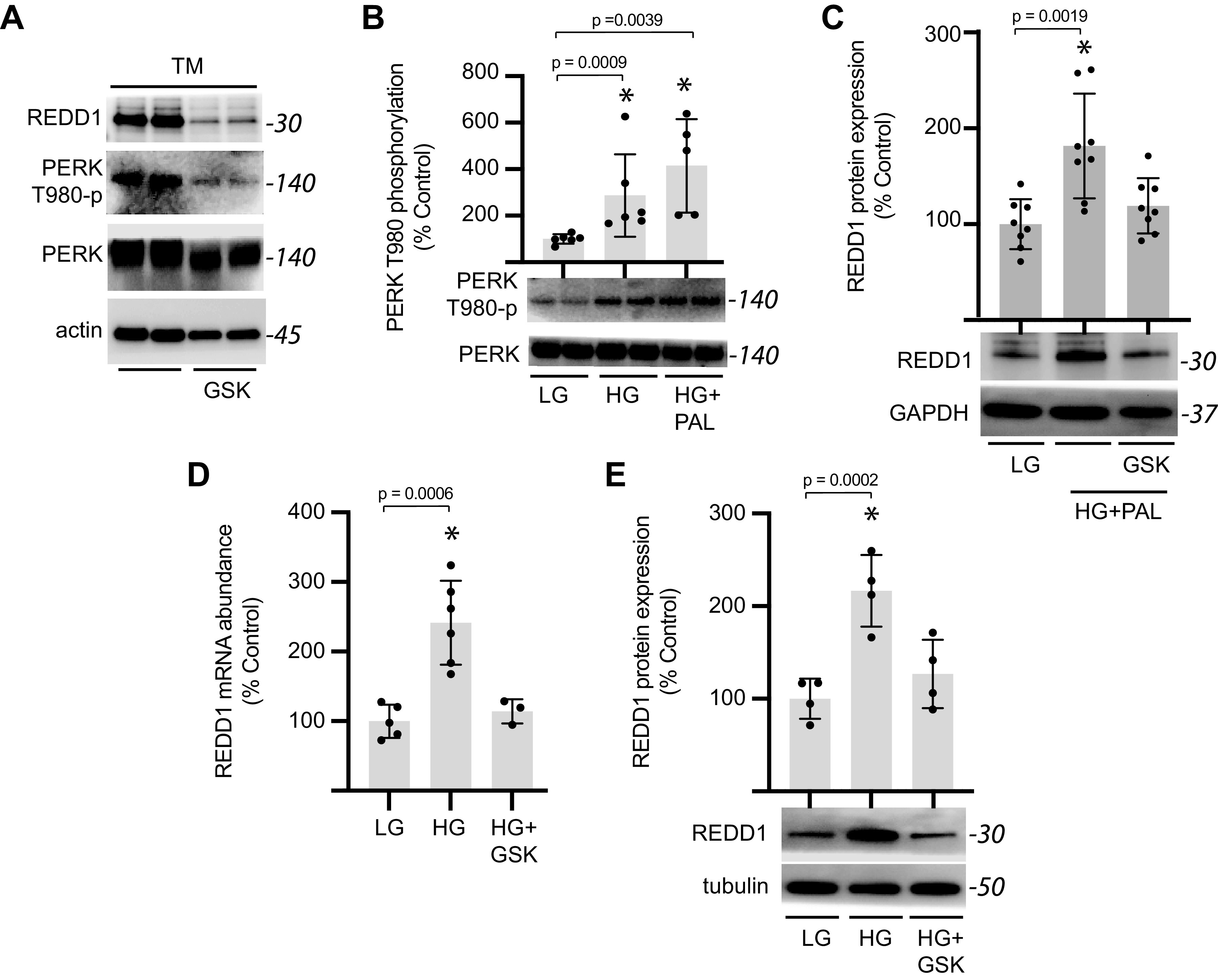
PERK activity is required for enhanced REDD1 expression in cardiomyocytes exposed to hyperglycemic/hyperlipidemic conditions. *A*: human AC16 cardiomyocytes were exposed to culture medium containing tunicamycin (TM) in the presence/absence of PERK inhibitor GSK2606414 (GSK) for 4 h. REDD1 expression and PERK phosphorylation at Thr980 were evaluated by Western blotting. Representative blots are shown. Protein molecular mass in kDa is shown at *right* of blots. *B*: AC16 cells were exposed to culture medium containing either 5 mM glucose (LG), 25 mM glucose (HG), or HG plus 0.5 mM BSA-conjugated palmitate (PAL) for 48 h. PERK phosphorylation and PERK expression were evaluated in cell lysates by Western blotting. *C*: Western blotting was used to evaluate REDD1 expression in AC16 cells exposed to HG and PAL for 48 h in the presence/absence of GSK. REDD1 mRNA (*D*) and protein (*E*) expression were evaluated in AC16 cells exposed to HG for 48 h in the presence/absence of GSK. Results are represented as means ± SD (*n* = 3–8 replicates). *P < 0.05 versus LG. PERK, protein kinase RNA-like endoplasmic reticulum kinase; REDD1, regulated in development and DNA damage 1.

To investigate a role for PERK in cardiomyocyte REDD1 expression, AC16 cells were exposed to the PERK inhibitor GSK2606414. GSK2606414 suppressed autophosphorylation of PERK at Thr980 in cardiomyocytes exposed to tunicamycin and attenuated REDD1 protein expression (Fig. 4*A*). Autophosphorylation of PERK was enhanced in cardiomyocytes exposed to hyperglycemic conditions in the presence or absence of palmitate (Fig. 4*B*), suggesting a potential role for PERK in the induction of REDD1 expression. Indeed, GSK2606414 reduced REDD1 expression in cardiomyocytes exposed to hyperglycemic/hyperlipidemic conditions (Fig. 4*C*). PERK inhibition prevented an increase in REDD1 mRNA (Fig. 4*D*) and protein (Fig. 4*E*) expression in cardiomyocytes exposed to hyperglycemic conditions.

### ATF4 Was Required for Enhanced REDD1 Expression in Cardiomyocytes Exposed to Hyperglycemic and Hyperlipidemic Conditions

**Figure 5.**
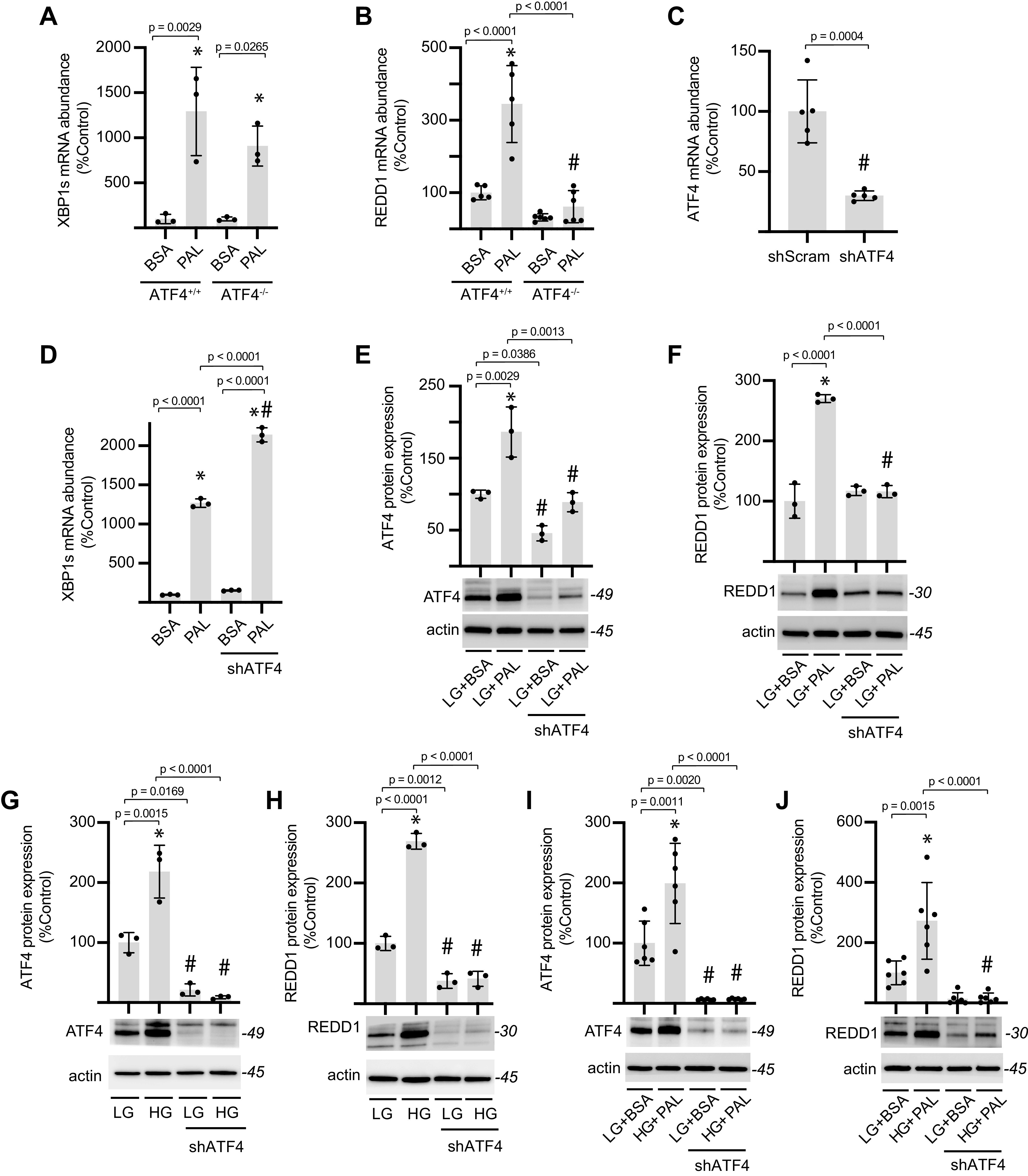
ATF4 is necessary for enhanced REDD1 expression in cardiomyocytes exposed to hyperglycemic or hyperlipidemic conditions. XBP1s (*A*) and REDD1 (*B*) mRNA expression were evaluated in ATF4^+/+^ and ATF4^−/−^ MEFs exposed to culture medium containing either 0.5 mM BSA-conjugated palmitate (PAL) or BSA alone for 8 h. *C*: ATF4 mRNA expression was knocked down in AC16 cells by stable expression of an shRNA targeting ATF4 (shATF4). ATF4 mRNA expression was evaluated in cells expressing shATF4 or a scramble shRNA control (shScram) by qPCR. AC16 cells were exposed to BSA or PAL for 24 h and XBP1s mRNA expression (*D*), ATF4 protein expression (*E*), and REDD1 protein expression (*F*) were evaluated by qPCR or Western blotting. Representative blots are shown. Protein molecular mass in kDa is shown at *right* of blots. AC16 cells were exposed to culture medium containing either 5 mM glucose (LG) or 25 mM glucose (HG) for 48 h and ATF4 (*G*) and REDD1 (*H*) protein expression was evaluated by Western blot. AC16 cells were exposed to culture medium containing either LG and BSA or HG and PAL for 48 h and ATF4 (*I*) and REDD1 (*J*) protein expression was evaluated by Western blot. Results are represented as means ± SD (*n* = 3–6 replicates). *P < 0.05 versus BSA, LG+BSA, or LG. #*P* < 0.05 versus ATF4^+/+^ or no shATF4. ATF4, activating transcription factor 4; MEF, mouse embryonic fibroblast; REDD1, regulated in development and DNA damage 1; XBP1, X-box DNA-binding protein 1.

To investigate a role for the transcription factor ATF4, ATF4^+/+^ and ATF4^−/−^ MEFs were exposed to culture medium supplemented with palmitate. Importantly, all MEFs were maintained in culture medium containing 25 mM glucose. XBP1 splicing was enhanced in both ATF4^+/+^ and ATF4^−/−^ MEFs exposed to palmitate (Fig. 5*A*). However, REDD1 expression was only enhanced in ATF4^+/+^, and not ATF4^−/−^ MEFs, exposed to hyperlipidemic conditions (Fig. 5*B*). To explore a role for ATF4 in cardiomyocytes, shRNA was used to knockdown ATF4 expression in AC16 cells (Fig. 5*C*). Enhanced abundance of XBP1s was observed in both control and shATF4-expressing cardiomyocytes upon exposure to medium containing palmitate (Fig. 5*D*). ATF4 (Fig. 5*E*) and REDD1 (Fig. 5*F*) protein expression was increased by exposure to medium containing palmitate. However, ATF4 knockdown reduced ATF4 expression and prevented an increase in REDD1 expression in cardiomyocytes exposed to hyperlipidemic conditions. AC16 cells exposed to hyperglycemic conditions also exhibited increased ATF4 (Fig. 5*G*) and REDD1 (Fig. 5*H*) protein expression. Moreover, ATF4 knockdown reduced the expression of both ATF4 and REDD1 and prevented an increase in the expression of either protein upon exposure to hyperglycemic conditions. ATF4 necessity was also observed for induction of ATF4 and REDD1 in cardiomyocytes exposed to both hyperglycemic conditions and palmitate (Fig. 5, *I* and *J*).

### REDD1 Contributed to Proinflammatory Cytokine Expression in Cardiomyocytes

**Figure 6.**
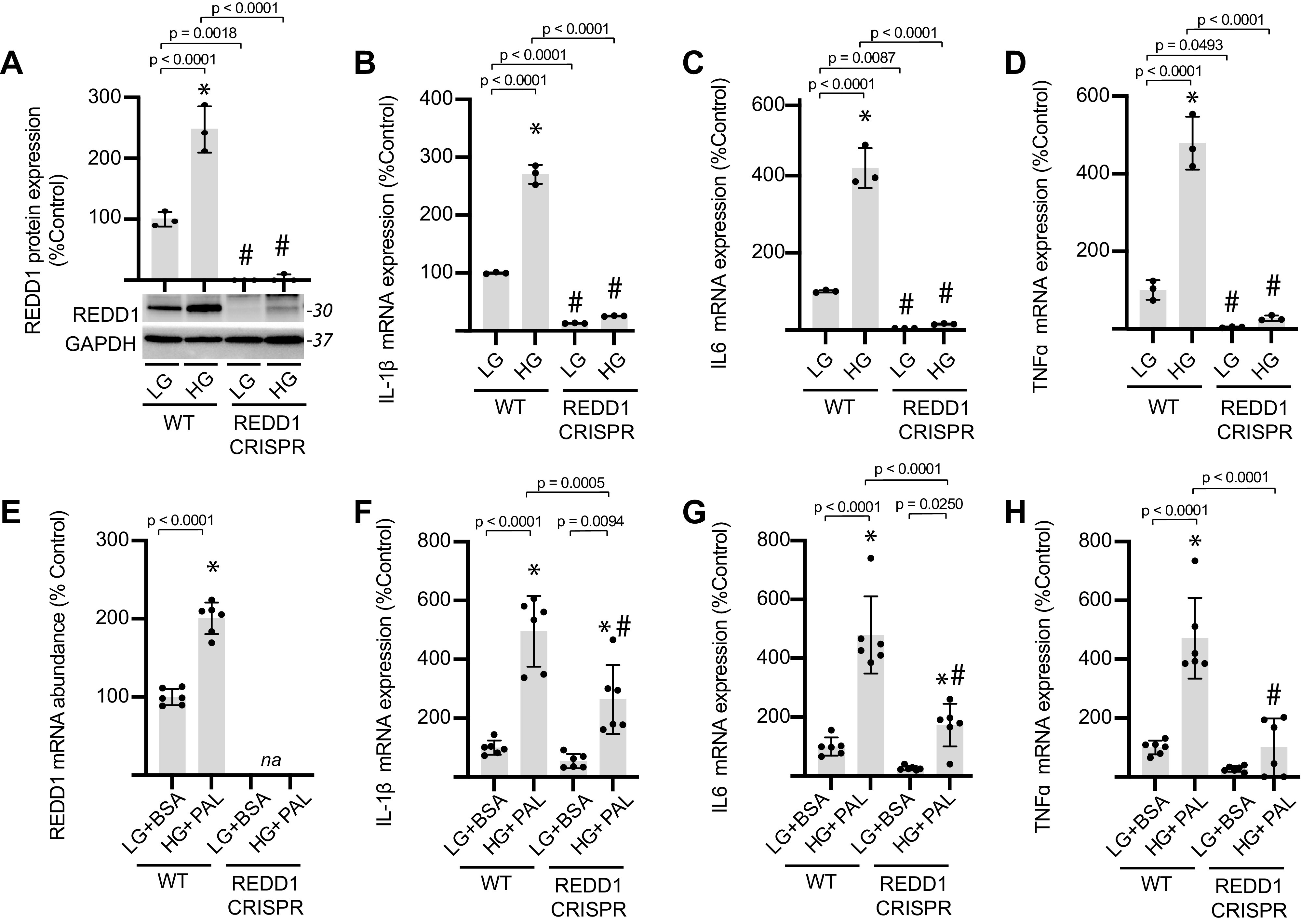
REDD1 contributes to proinflammatory cytokine expression in cardiomyocytes. *A*–*D*: wild-type (WT) and REDD1 CRISPR AC16 cardiomyocytes were exposed to medium containing either 25 mM glucose (HG) or 5 mM glucose with 20 mM mannitol as an osmotic control (LG) for 48 h. REDD1 protein expression was evaluated by Western blotting. Representative blots are shown. Protein molecular mass in kDa is shown at *right* of blots. *E* and *F*: WT and REDD1 KO AC16 cardiomyocytes were exposed to medium containing either 0.5 mM BSA-conjugated palmitate (PAL) and HG or BSA alone for 48 h. Expression of mRNAs encoding IL-1β (*B* and *E*), IL6 (*C* and *F*), and TNFα (*D* and *G*) was evaluated in cell lysates by qPCR. Results are represented as means ± SD (*n* = 3–6 replicates). *P < 0.05 versus LG or LG+BSA; #*P* < 0.05 versus WT. na, not assessed. REDD1, regulated in development and DNA damage 1.

**Figure 7.**
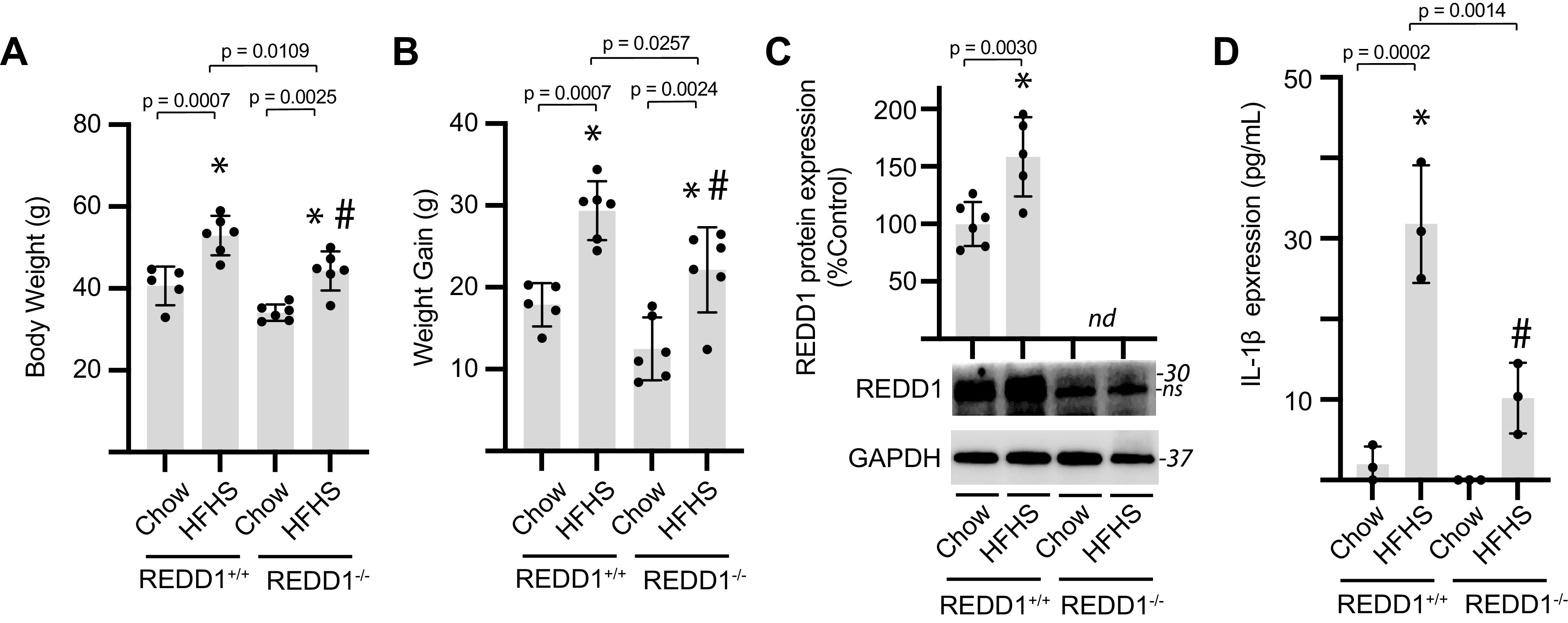
REDD1 deletion reduces IL-1β expression in the heart of mice fed a HFHS diet. *A*–*D*: wild-type (REDD1^+/+^) and REDD1 knockout (REDD1^−/−^) mice were fed either a HFHS diet or control chow diet for 16 wk. Body weight (*A*) and weight gain (*B*) were determined. *C*: REDD1 expression was evaluated in cardiac tissue homogenates by Western blotting. Representative blots are shown. Protein molecular mass in kDa is shown at *right* of blots. nd, not determined; ns, non-specific. *D*: IL-1β protein expression in cardiac tissue homogenates was determined by ELISA. Results are represented as means ± SD (*n* = 3–6 mice). *P < 0.05 versus chow. #*P* < 0.05 versus wild type. HFHS, high-fat high-sucrose; REDD1, regulated in development and DNA damage 1.

REDD1 protein expression was increased in AC16 cardiomyocytes exposed to hyperglycemic conditions, and REDD1 deletion was sufficient to prevent the effect (Fig. 6*A*). In cardiomyocytes exposed to hyperglycemic conditions, we observed a REDD1-dependent increase in expression of the inflammatory markers IL-1β (Fig. 6*B*), IL-6 (Fig. 6*C*), and TNFα (Fig. 6*D*). Similarly, exposure to hyperglycemic conditions and palmitate enhanced the abundance of mRNAs encoding REDD1 (Fig. 6*E*), IL-1β (Fig. 6*F*), IL-6 (Fig. 6*G*), and TNFα (Fig. 6*H*); and REDD1 deletion was sufficient to reduce the expression of inflammatory markers in cardiomyocytes exposed to hyperglycemic/hyperlipidemic conditions. To determine if REDD1 played a similar role in the heart, REDD1-deficient mice were fed an HFHS diet for 16 wk. REDD1^+/+^ and REDD1^−/−^ mice fed an HFHS diet exhibited increased body weight (Fig. 7*A*) and enhanced weight gain (Fig. 7*B*) as compared with littermates fed a chow diet. In heart homogenates from REDD1^+/+^ mice fed an HFHS diet, expression of REDD1 (Fig. 7*C*) and IL-1β (Fig. 7*D*) was increased as compared with littermates fed a control chow diet. As compared with REDD1^+/+^ mice fed an HFHS diet, IL-1β expression was reduced in heart homogenates from REDD1^−/−^ mice fed an HFHS diet.

## DISCUSSION

Overnutrition and sedentary lifestyle are major contributing factors to the worldwide epidemic of obesity and type 2 diabetes. Clinical studies demonstrate that consumption of a diet rich in saturated fats and refined carbohydrates is predictive of cardiovascular disease (30, 31). As compared with mice fed a standard chow diet, mice fed an HFHS diet exhibited accelerated weight gain within 1 wk and increased plasma insulin and lipid levels by 2 wk (32). After 8 wk of HFHS diet, mice exhibit evidence of both impaired systolic and diastolic function (33). In the present study, we evaluated the impact of an HFHS diet on cardiomyocyte REDD1 expression. In both, the hearts of mice fed an HFHS diet and in cardiomyocyte cultures exposed to hyperglycemic/hyperlipidemic conditions, REDD1 expression was enhanced coincident with markers of ER stress and inflammation. Overall, the findings are consistent with a model wherein the HFHS diet contributes to the development of myocardial inflammation by promoting REDD1 expression via activation of a PERK/ATF4 signaling axis.

ER stress has been shown in preclinical rodent models of both type 1 and type 2 diabetes, as well as in the myocardium of humans with diabetes (7, 8, 34). Consistent with prior reports, we observed increased XBP1 processing and enhanced ATF3 expression in the myocardium of mice fed an HFHS diet for 12 wk. A prior study demonstrated that the eIF2α kinase PERK was required for ER stress-induced REDD1 expression in MEFs exposed to tunicamycin (13). Herein, we found a similar role for the kinase in cardiomyocytes, as REDD1 expression in cells exposed to tunicamycin was reduced when PERK activity was inhibited. Similarly, PERK inhibition prevented the upregulation of REDD1 expression in cardiomyocytes exposed to hyperglycemic/hyperlipidemic conditions.

The initiation step of mRNA translation requires the assembly of an eIF2 ternary complex, consisting of eIF2, GTP, and met-tRNA_i_ (35). Each round of translation initiation produces eIF2-GDP, which must be recycled to eIF2-GTP in a reaction catalyzed by the guanine nucleotide exchange factor (GEF) eIF2B. Phosphorylation of the eIF2α subunit on S51 results in tighter binding to eIF2B, effectively converting eIF2α into a competitive inhibitor of GEF activity to repress eIF2 ternary complex formation (36). As a result, global rates of mRNA translation are inhibited; however, translation of a small number of mRNAs is paradoxically enhanced by eIF2α phosphorylation. Stress response proteins such as ATF4 are encoded by mRNAs containing multiple upstream open reading frames in their 5′-untranslated regions. When eIF2 ternary complex is abundant, these sequence elements act as an impediment to scanning and identification of the proper start codon. However, when ternary complex availability is limited, the translation of mRNAs with multiple upstream open reading frames is maintained or even enhanced (12). In the present study, ATF4 protein expression was increased in cardiomyocytes exposed to hyperglycemic conditions. Moreover, ATF4 knockdown was sufficient to prevent increased expression of REDD1 in response to hyperglycemic or hyperlipidemic conditions.

Myocardial inflammation is believed to play a critical role in the development and progression of heart failure (37). Inflammation is a protective immune response designed to facilitate tissue repair. However, when the inflammatory response is not properly regulated, it may lead to chronic inflammation and disease pathogenesis. In mice fed a high-fat diet, myocardial inflammation is evidenced by enhanced expression of proinflammatory cytokines (38). In support of the prior work, IL-1β expression was increased in the myocardium of mice fed an HFHS diet. The studies here expand on the prior report by demonstrating a role for REDD1 in the expression of inflammatory cytokines by cardiomyocytes. Expression of IL-1β, as well as IL-6, and TNFα, were enhanced in cardiomyocytes exposed to hyperglycemic/hyperlipidemic conditions coincident with an increase in REDD1 expression. REDD1 deletion reduced the expression of proinflammatory cytokines in cardiomyocytes exposed to hyperglycemic/hyperlipidemic culture conditions. Furthermore, cardiovascular tissue of REDD1-deficient mice fed an HFHS diet exhibited reduced IL-1β expression as compared with wild-type. A role for REDD1 in cardiac inflammation supports a prior report demonstrating reduced inflammatory cytokine expression in a model of doxorubicin-induced cardiomyopathy upon REDD1 knockdown (25).

Overall, the findings support the key role of REDD1 in activation of inflammatory pathways (39–42). REDD1 is best known as an inhibitor of mechanistic target of rapamycin complex 1 (mTORC1)/Akt (43) but has more recently been shown to act independently of mTORC1/Akt signaling to regulate the transcription factor nuclear factor kappa B (NF-κB) (39). REDD1 acts to enhance NF-κB signaling by directly sequestering inhibitor of κB (IκB), and thus facilitates nuclear localization of the transcription factor (39). NF-κB stimulates the production of proinflammatory cytokines, acute phase proteins, and chemokines, and is enhanced in models of cardiac dysfunction (44, 45). NF-κB activation is observed in the hearts of diabetic rats (46) and in cardiomyocytes exposed to hyperglycemic conditions (47). Studies also support a role for hyperlipidemia and elevated plasma lipoproteins in NF-κB activation (48). Thus, it is tempting to speculate that the suppressive effect of REDD1 deletion on expression of IL-1β in the heart of mice fed an HFHS diet may be driven by blunted NF-κB activation.

An important caveat to the data herein is that the REDD1 knockout mouse model exhibits whole body REDD1 deletion. Thus, it is not possible to conclude that the suppressive effect of REDD1 deletion on IL-1β expression in heart tissue homogenates is directly due to REDD1 suppression in cardiomyocytes per se, as systemic factors may also contribute to the effect. Indeed, REDD1^−/−^ mice fed an HFHS diet exhibited a modest reduction in body weight and weight gain as compared with REDD1^+/+^ mice fed an HFHS diet. This likely reflects a reduction in circulating insulin concentrations in the REDD1^−/−^ mice (49) and suggests the possibility that systemic factors contribute to reduced IL-1β expression in the heart of REDD1-deficent mice fed an HFHS. However, REDD1 deletion was sufficient to suppress IL-1β, IL-6, and TNFα expression in cardiomyocyte cultures exposed to hyperglycemic/hyperlipidemic conditions. Together, the data are consistent with a working model wherein REDD1 expression in cardiomyocytes contributes to proinflammatory cytokine expression.

Diabetes is recognized as a state of chronic inflammation characterized by increased production of inflammatory cytokines, which contribute to the development of cardiovascular complications. Drugs that antagonize the actions of IL-1β have demonstrated benefits in reducing cardiovascular events in patients with prior myocardial infarction (50). Herein, REDD1 deletion reduced IL-1β expression in the hearts of mice fed a prodiabetogenic diet, and similar benefits were observed with REDD1 deletion in human cardiomyocytes exposed to hyperglycemic/hyperlipidemic culture conditions. These proof-of-concept studies support the possibility that therapeutic intervention to suppress REDD1 expression in cardiomyocytes may offer an alternative for reducing the production of IL-1β, as well as other inflammatory cytokines.

## SUPPLEMENTAL DATA

10.6084/m9.figshare.21420399 Supplemental Tables S1 and S2 and Figure S1: https://doi.org/10.6084/m9.figshare.21420399.

## GRANTS

This research was supported by the National Institutes of Health grants HL165924 (to S.A.S.), EY029702, EY032879 (to M.D.D.), and DK015658 (to S.R.K.). M.K.G.A. was supported by an American Heart Association Undergraduate Research Fellowship (898925).

## DISCLOSURES

No conflicts of interest, financial or otherwise, are declared by the authors.

## AUTHOR CONTRIBUTIONS

S.A.S., J.P., E.A.B., and M.D.D. conceived and designed research; S.A.S., M.K.G.A., A.L.T., E.I.Y., S.S., and A.M.V. performed experiments; S.A.S., A.L.T., S.S., and M.D.D. analyzed data; S.A.S., S.S., J.P., S.R.K., and M.D.D. interpreted results of experiments; S.A.S. and M.D.D. prepared figures; S.A.S. and M.D.D. drafted manuscript; S.A.S., A.L.T., E.I.Y., S.S., A.M.V., J.P., E.A.B., S.R.K., and M.D.D. edited and revised manuscript; S.A.S., M.K.G.A., A.L.T., E.I.Y., S.S., A.M.V., J.P., E.A.B., S.R.K., and M.D.D. approved final version of manuscript.
